# Perforation of the Ileum1Read at the Illinois State Veterinary Medical Association, February 15, 1899.

**Published:** 1899-06

**Authors:** C. E. Hollingsworth

**Affiliations:** La Salle, Ill.


### PERFORATION OF THE ILEUM.[1]

BY C. E. HOLLINGSWORTH, V.S.,

LA SALLE, ILL.

AT 7 A.M. on Sunday, February 13, 1898, I was called to attend a seven-year-old bay mare of 1300 pounds weight, which was highly fed and in good flesh, and for a year past had been used in a coal team. There had been no previous sickness in all this time, and at 3 A.M. she was comfortably eating hay.

Profuse diarrhœa was present; pulse 65 and slightly sthenic; accelerated respiration, and with the nose kept almost constanty at the bottom of the manger. Rigors of a very pronounced character, with other symptoms, caused me to examine the lungs, but with a negative result; also, to take the temperature, which registered $104\frac{3}{5}$° F. Once or twice she attempted to lie down, but before getting quite down would rise again. There was no tympanites,

[1] Read at the Illinois State Veterinary Medical Association, February 15, 1899.

and no severe pain indicative of indgestion or colic in any form. She simply stood there, trembling all over, with rapid breathing and distended nostrils, every few moments passing small quantities of liquid feces mixed with mucus, each evacuation of which intensified the rigors. The extremities were cold, and food and drink refused ; a very anxious expression was noticeable.

I scarcely knew what name to give to the disease, but told the owner the trouble was in the posterior portion of the bowels. With such a variety of symptoms and conditions I recognized it as a very unusual case—at least to me. I was not positive of the exact nature of it, consequently my treatment was not very radical, but more of an expectant nature. I gave a dose of sedative medicine and stayed an hour to watch the effects ; also had her well blanketed. Within half an hour the rigors were almost gone, the surface of the body was warm, and the patient appeared easier in every respect. Thinking possibly I was making a "mountain out of a mole-hill," I left a small dose of fluid extract of veratrum viride and a full dose of fluid extract of belladonna and fluid extract of nux vomica, to be given an hour later.

Returning at 10 A.M. I found the symptoms about the same in general, except that the feces were not passed so frequently; rigors had returned, but in a slight degree, and there was now some perspiration ; the head persistently held down in the manger. The urine was passed fully an hour before my return for the first time, and frequent attempts to urinate were made afterward with some straining. I discontinued the sedative, but kept on with the fluid extract of belladonna, adding fluid extract of cannabis indica.

On returning at 3 P.M. I found every symptom greatly increased except the diarrhœa ; the pulse 100, temperature 108° F., profuse perspiration, and rigors of the worst type. My mental prognosis was a rapidly fatal termination, but I left small doses of acetanilid to be given every two hours.

On returning at 9.30 P.M. I found the mare had been dead only a short time.

Autopsy, next morning, revealed a perforation of the ileum, one-half inch in diameter, and about a pint of fecal matter had escaped and was lodged between the folds of the mesentery. Traces of liquid feces were discernible all over the abdominal cavity, and extensive inflammation throughout the posterior bowels; also the lungs and kidneys were slightly inflamed—the result of the morbid conditions. All the other organs were normal. What caused the perforation ?

